# Elevated CSF histamine levels in multiple sclerosis patients

**DOI:** 10.1186/2045-8118-10-19

**Published:** 2013-05-09

**Authors:** Ulf Kallweit, Kosuke Aritake, Claudio L Bassetti, Stephan Blumenthal, Osamu Hayaishi, Michael Linnebank, Christian R Baumann, Yoshihiro Urade

**Affiliations:** 1 Department of Neurology, University Hospital Zurich, Frauenklinikstrasse 26, 8091 Zurich, CH, Switzerland; 2 Department of Molecular Behavioral Biology, Osaka Bioscience Institute, Osaka, Japan; 3 Department of Anesthesiology and Intensive Medicine, Triemli Hospital, Zurich, CH, Switzerland

**Keywords:** Multiple sclerosis, Histamine, Histamine receptors, CSF, Granulocyte-macrophage colony-stimulating factor, Chronic inflammatory diseases

## Abstract

**Background:**

Histamine is an ubiquitous inflammatory mediator of numerous physiological processes. Histamine and its receptors have been implicated in multiple sclerosis (MS) disease pathogenesis. We prospectively enrolled 36 MS patients and 19 age and gender-matched healthy volunteers for cerebrospinal fluid (CSF) histamine analysis.

**Findings:**

CSF histamine levels in MS patient samples were significantly higher (median: 35.6 pg/ml) than in controls (median: 5.5 pg/ml; Beta = 0.525, *p* < 0.001). In addition, histamine increased with age (Pearson’s correlation, *p* < 0.003).

**Conclusions:**

Histamine may be an important factor for both the initiation and maintenance of chronic inflammatory diseases of the central nervous system. Our observation encourages a deeper investigation of the role of histamine in MS.

## Background

Multiple sclerosis (MS) is a complex autoimmune disease with inflammation and demyelination within the central nervous system (CNS). Histamine and its receptors have been implicated in MS disease pathogenesis [1]. Histamine is an ubiquitous inflammatory mediator of numerous physiological processes including local immune responses and allergic reactions, and it plays an important role in neurotransmission. The functions of histamine are mediated through 4 G-protein coupled receptors (H1-H4 receptors). Histamine and histamine receptors play a regulatory role in experimental allergic encephalomyelitis (EAE), the animal model of MS [1-3]. Rodent experiments revealed that histamine increases the permeability of the blood brain barrier, promoting CNS inflammation [1,4,5]. The cytokine granulocyte-macrophage colony-stimulating factor (GM-CSF) in auto-reactive T helper cells is mandatory to induce and sustain EAE in mice [6]. GM-CSF stimulates granulocytes and macrophages, and both secrete histamine. A small study by Tuomisto et al. [7], reported on higher CSF histamine level by about 60% both in patients with remitting and progressive type of MS than in controls. Based on this observation and previous studies in EAE, we hypothesized that histamine might play a role in MS. Thus, we aimed at testing whether histamine is elevated in the CSF of MS patients.

## Findings

### Methods

We prospectively enrolled consecutive 36 MS patients (mean (±SD) age, 51 ± 10 years; 64% female; 24 patients with secondary progressive disease, 12 relapsing-remitting, RRMS) of Caucasian origin treated in the MS center of the University Hospital Zurich and 19 age and gender-matched healthy volunteers: age 46 ± 10 years (mean ±SD); 37% female. In all patients, the diagnosis of MS was made according to the McDonald diagnostic criteria 2005 [8]. We enrolled all types of MS. Lumbar puncture was performed for diagnostic purposes. In 19 patients, LP was performed for diagnosis of MS and in 17 secondary progressive MS (SPMS) patients for evaluation of treatment effects of first-time intrathecal baclofen (N = 7) or triamcinolone acetonid (N = 10) application. In the controls, cerebrospinal fluid (CSF) was ascertained during spinal anesthesia. Subjects with signs of neurological or sleep-wake disorders were not included as controls. Patients and controls suffering from any allergic disease were not included into this study. All patients and healthy controls gave informed written consent. The study was approved by the local ethics committee.

Routine CSF analysis included oligoclonal bands and IgG index. For histamine analysis, after assessment of CSF, samples were immediately centrifuged, put on dry ice and stored at −80°C until assay. Storage was between 6 and 24 months (patients and controls). As far as we examined, histamine contents in biological samples stored at −80°C did not change over 2 years. Samples were separated by high-performance liquid chromatography (HPLC), thereafter histamine was post-labeled by O-phthalaldehyde and detected by fluorometric analysis [9]. HPLC has been used for the measurement of histamine in CSF in other neurological disorders [10].

Statistical analyses were performed with SPSS 19.0. Group data are described by median. For the comparison of parametric data, we Student’s t-tests, and for non-parametric data, Whitney *U*-test was used. For correlation analyses in parametric data, we used Pearson’s correlation. To control for effects of sex, age and different types of MS on histamine concentrations, we ran multivariate logistic regression analyses.

### Results

**Figure 1:**
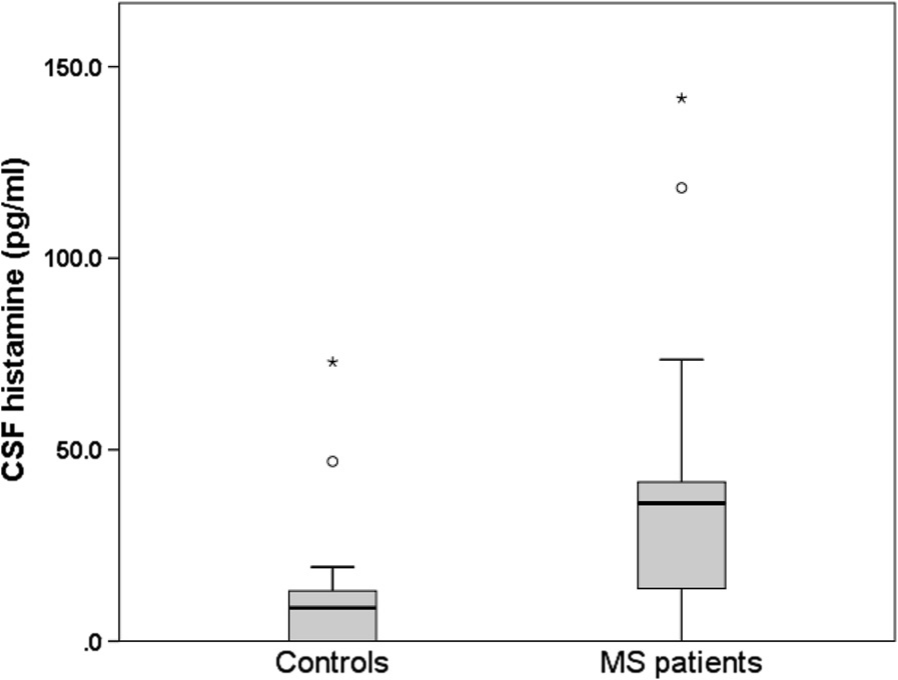
**Boxplot of cerebrospinal fluid histamine levels in 36 patients with MS and in 19 healthy controls.** The horizontal solid line depict median levels per group. The bottom of the box represents the 25^th^ percentile, the top of the box the 75^th^ percentile. T-bars represent the lowest datum still within 1.5 interquartile range (IQR) of the lower quartile, and the highest datum still within 1.5 IQR of the upper quartile. Dots and stars represent extreme outliers (each symbol represents one patient).

RRMS patients received disease modifying therapies (DMT): Interferon-beta 1a- i.m. (Avonex, N = 4), Interferon-beta 1a, s.c. (Rebif, N = 4), Glatirameracetat s.c. (Copaxone, N = 3), Natalizumab 300 mg i.v. (Tysabri, N = 1). In SPMS patients, 4 patients received DMT (Interferon-beta 1a, s.c. (Rebif, N = 2), Mitoxantrone i.v. (N = 2). Previously all SPMS patients were treated by at least one DMT. CSF histamine levels in patient samples are shown in the boxplot in Figure 1. After exclusion of the two each outliers, histamine levels in patients (median: 35.6 pg/ml) were significantly higher than in controls (median: 5.5 pg/ml; Beta = 0.525, *p* < 0.001). Also without exclusion of outliers, this difference was significant (*p* < 0.017). In addition, histamine increased with age (Pearson’s correlation, *p* < 0.003), but were similar in men (30.0 ± 27.0) and women (39.2 ± 6.4; t = 0.89; *p* < 0.381) and were not associated with diagnosis of relapsing remitting (26.0 ± 28.2) versus secondary progressive MS (39.9 ± 31.6; t = 1.5; *p* < 0.154). No correlations were found between CSF histamine levels and the results of other CSF analyses, (e.g. IgG index), treated and non-treated patients or existing DMT (data not shown).

## Discussion

This study confirmed results by Tuomisto [7] and our hypothesis that histamine levels are increased in MS patients. Histamine functions are not only limited to allergic functions but are extended to other processes related to neurology, immunology, or oncology [11]. Until now, no CNS disease entity has been associated directly with brain histamine dysfunction, but different histamine receptors [H(3), H(4)] are recognized as drug targets for (e.g.) sleep-wake disorders, Parkinson’s disease, and neuroimmunopharmacology [12].

Histamine in CSF originates from mast cells and histaminergic neurons of the tuberomammillary nucleus of the posterior basal hypothalamus [13]. The GM-CSF-mediated stimulation of granulocytes and macrophages may be one possible underlying mechanism for elevated histamine levels in MS. Thus histamine may be an important factor for both the initiation and maintenance of chronic inflammatory diseases of the central nervous system such as MS. This interpretation remains theoretical until data from other patient groups with acute and chronic inflammatory and non-inflammatory diseases of the CNS confirm our results.

Limitations of our study include the small study size and the large intra-group variability of histamine levels. We did not measure histamine levels in blood. Although peripheral histamine hardly enters into CNS through blood brain barrier [14,15], we cannot completely exclude the possibility that histamine concentrations in CSF are not only controlled in brain, but also derive from blood. Furthermore, we cannot exclude DMT effects on histamine levels.

## Conclusions

This observation encourages a deeper investigation of the role of GM-CSF, granulocytes, macrophages and histamine in MS. Further, histamine may be investigated as diagnostic marker for MS and other inflammatory CNS diseases.

## Competing interest

The Authors declare that there is no conflict of interest.

## Authors’ contributions

Conception and design: UK, KA, CLB, OH, CRB, YU. Acquisition of data: UK, SB, ML, CRB. Analysis and interpretation of data: UK, KA, CLB, ML, YU. Drafting the manuscript: UK. Revising it critically for important intellectual content: KA, CLB, SB, OH, ML, CRB, YU. All authors have given final approval of the version to be published.
